# Cystatin C-derived estimated glomerular filtration rate in children with sickle cell anaemia

**DOI:** 10.1186/s12882-023-03393-x

**Published:** 2023-11-29

**Authors:** Hakeem Edun Babatunde, Afeez Oyesola Bello, Muhammed A. Nurudeen Adeboye, Olumuyiwa Shola Folayan, Olugoke Ezekiel Ojewole, Usman Abubakar

**Affiliations:** 1 Department of Disease Control and Elimination, Medical Research Council Unit, The Gambia at London School of Hygiene and Tropical Medicine, Atlantic Boulevard, P. O. Box 273, Banjul, The Gambia; 2 Department of Paediatrics, Federal Medical Centre, Bida, Niger State Nigeria; 3 Department of Paediatrics And Child Health, University Of Ilorin/UITH, Ilorin, Kwara State Nigeria; 4 Department of Paediatrics, University College Hospital, Ibadan, Nigeria

**Keywords:** Children, Cystatin C, Glomerular filtration rate (GFR), Hyperfiltration, Sickle cell anaemia, Decreased GFR

## Abstract

**Background:**

Sickle cell disease is the most common inherited blood disorder in humans and constitutes a major public health burden. It is a multisystemic condition with long-term renal complications. Early detection of sickle cell nephropathy and initiation of appropriate interventions are associated with improved survival and quality of life. This study aimed to compare the cystatin C-derived estimated glomerular filtration rate (GFR) of the study groups and also, to correlate the clinical features of chronic kidney disease (CKD) with decreased GFR in children with sickle cell anaemia (SCA).

**Methods:**

This hospital-based cross-sectional analytic study recruited 86 SCA subjects in steady-state and 86 age and sex-matched healthy HbAA controls aged 1–14 years who attended the Paediatric Haematology and Outpatient clinics of Federal Medical Centre Bida over six months. Data were collected using a semi-structured questionnaire, and participants’ length/height, weight, and blood pressure were measured using standard procedures. Blood samples were drawn for serum cystatin C assay via the sandwich enzyme-linked immunosorbent assay (ELISA) technique. Filler’s equation was used to calculate the glomerular filtration rate.

**Results:**

There was a significant difference in the mean cystatin C-derived GFR between the two groups, i.e. 116 ± 30mL/min/1.73m^2^ vs. 106 ± 24mL/min/1.73m^2^ for the SCA and control groups, respectively (p = 0.017). The prevalence of supernormal GFR (i.e. GFR > 140mL/min/1.73m^2^) and decreased GFR (i.e. GFR < 90mL/min/1.73m^2^) was 19.8% and 22.1%, respectively, in children with SCA. There was no significant association between the age at diagnosis of SCA, blood transfusions, blood pressure, packed cell volume and presence of peripheral oedema with decreased GFR in the study subjects.

**Conclusions:**

Supernormal GFR is common in children with SCA and there is no significant association between clinical features of CKD with decreased GFR. Regular evaluation of renal function is, however, recommended in children with SCA for early detection and treatment of renal complications in order to halt the progression to end-stage kidney disease (ESKD).

## Background

Sickle cell disease (SCD) is the most common inherited blood disorder in humans and was originally restricted to people occupying malaria-endemic regions of the world [1, 2]. Due to widespread international migration, however, cases are now found across the world [2, 3]. It is a chronic and often life-threatening disorder in which red blood cells assume a sickle shape. It results from the inheritance of two abnormal haemoglobin genes, at least one of which is responsible for sickle haemoglobin (HbS) formation. The most common and severe clinical phenotype is homozygote disease (HbSS), also referred to as sickle cell anaemia (SCA) [4].

Sickle cell disease is prevalent in the tropics with the largest burden in sub-Saharan African countries of Nigeria and the Democratic Republic of Congo [4]. More than 300,000 babies are born with SCD worldwide yearly, the majority of whom die before their fifth birthday due to bacterial sepsis, severe anaemia and suboptimal care [4, 5]. Nigeria has an SCA birth incidence of 2% and a carrier state (HbAS) ranging from 20–30% [6]. Similarly, approximately 91,000 of Nigeria’s newborns had SCA in 2010 and are projected to be more than 140,000 by the year 2050 [4]. Therefore, SCD constitutes a major health burden in the West African subregion where Nigerians account for about half of the population [6].

Sickle cell anaemia is a multisystemic illness with manifestations of acute illness and progressive organ damage due to vascular obstruction from sickled red cells. The kidney is affected in several ways (generally referred to as sickle cell nephropathy [SCN]) and is a common cause of morbidity and mortality. The absence of a comprehensive paediatric renal registry in most sub-Saharan African countries suggests that the true burden of kidney disease is largely unknown. Nevertheless, it is said to account for 16–18% of mortality among patients with SCD [7]. Furthermore, as advancement in clinical care promotes the survival of these patients into adulthood, SCN imposes a growing burden on both individual health and health system costs.

Sickle cell nephropathy manifests during early childhood. As such, there has been a rising interest in its prevention, detection and treatment. The lack of neonatal screening programmes in most African settings has caused significant delays in SCA diagnoses. Serum cystatin C is a highly sensitive renal biomarker that is influenced less by demographic variables such as age, sex and muscle mass [8, 9]. Unlike serum creatinine, the cystatin C level is not affected by the presence of chromogens such as bilirubin and haemoglobin in blood samples, and its clearance is not affected by proximal tubular hyperactivity which are preeminent features in SCD [10]. This study set out to compare the cystatin C-derived estimated glomerular filtration rate (GFR) of the study groups and also, to correlate the clinical features of chronic kidney disease (CKD) with decreased GFR in children with sickle cell anaemia.

## Methods

### Study design

This was a hospital-based, cross-sectional analytic study.

### Study setting

This study took place in the Paediatric Haematology and Outpatient clinics of the Federal Medical Centre Bida, North-Central Nigeria. Bida, an ancient town of the Nupe kingdom, is situated at latitude 9.08^0^ North, longitude 6.02^0^ East and 151 m above sea level. The study participants were recruited over six months, i.e. from October 2019 to March 2020, and blood samples were analysed for serum cystatin C assay at the chemical pathology laboratory of the institution.

### Study population

The study participants consisted of children with SCA and their age and sex-matched HbAA control age range of 1–14 years via a one-to-one pairing system. Children with SCA were selected based on the documented result of haemoglobin electrophoresis from the Paediatric Haematology Clinic. The controls were children on follow-up visits at the paediatric medical/surgical outpatient clinics discharged from the hospital for ailments such as malaria, pneumonia, asthma, cerebral palsy and minor surgical procedures. Patients who had previously been diagnosed with kidney disease, cardiac disease, HIV/AIDS, or those on systemic steroids were excluded from participation. The participants were selected by purposive sampling and enrolled consecutively in the study until each group’s desired sample size was achieved.

### Sample size consideration

The study sample size of 172 participants comprised 86 children with SCA in steady-state (defined as the period free of crisis extending from at least three weeks since the last clinical event and three months since the last blood transfusion) and 86 healthy age- and sex-matched HbAA controls. This was achieved using a standard formula for comparing two independent group means and setting the study power at 90%. Data on eGFR in children with SCA from a published study were used in the calculation to derive our sample size [11].

### Data collection

Relevant sociodemographic and clinical data were collected using a semi-structured questionnaire. The participants’ length/height, weight, and blood pressure were measured using standard procedures. Whereas the participants’ blood pressure was interpreted according to the updated American Academy of Pediatrics Clinical Practice Guidelines for Screening and Management of High Blood Pressure in Children and Adolescents [12], the body mass index (BMI) was interpreted via the World Health Organization BMI-for-age charts [13]. Data on socioeconomic class were obtained by the method described by Oyedeji [14].

### Blood sample collection

Blood was drawn from a venepuncture site using an aseptic technique. The haemoglobin phenotype of the participants was ascertained using cellulose acetate paper electrophoresis at a pH of 8.6. Two millilitres of blood were emptied into a plain bottle, left to clot at room temperature, and then centrifuged at 1000 rpm for 15 min. The resultant serum was transferred into a separate plain bottle and stored at -80 °C for the cystatin C assay.

### Sample analysis

Quantitative analysis of cystatin C was carried out via the sandwich enzyme-linked immunosorbent assay (ELISA) principle. Two 96-well kits manufactured by Elabscience® Biotechnology Inc., Texas, one each for the two groups of participants, were used in this study. The ensuing product of the sandwich was read spectrophotometrically at 450 nm using a TC-96 micro-well reader (Teco Diagnostics, California). The estimated glomerular filtration rate (eGFR) in mL/minute/1.73m^2^ was calculated using a cystatin C-based equation by Filler and Lapage as its diagnostic performance was validated in children aged 1–18 years and is represented thus [15]: Log(GFR) = 1.962 + [1.123 × log(1/cystatin C)]. The cutoff value of < 90mL/min/1.73m^2^ was used to define decreased GFR in SCA subjects because the standard definition of CKD (i.e. GFR < 60mL/min/1.73m^2^) may represent a greater decline from “normal” kidney function in SCA patients when compared to the general population.

### Data analysis

The data obtained were verified and statistical analysis was carried out using the Statistical Package for Social Sciences version 28.0 software (SPSS; Chicago, USA). Categorical variables are presented in tables. Upon ascertainment of data normality, the means of continuous variables were compared using an *independent t test*. Relationships between categorical and continuous variables were ascertained using the *Chi-square* or *Exact test*, as appropriate. Using Python programming language with its data science ecosystem (PyData Stack; Texas, USA), we calculated the uncertainty coefficient and correlation ratio between variables as well as built models to predict decreased eGFR from clinical and demographic data. A *p* value of ≤ 0.05 was considered statistically significant.

## Results

### General characteristics of the participants

The sociodemographic distribution, medical history and physical examination findings of the study participants are depicted in Table 1. Most of the children with SCA were diagnosed after their first birthday, and the majority had multiple crises in the year preceding enrollment.

**Table 1:** General characteristics of the study participants

Variable	Subject (%)	Control (%)
Age (years)		
1–5	30 (34.9)	30 (34.9)
6–10	38 (44.2)	38 (44.2)
>10	18 (20.9)	18 (20.9)
Sex		
Male	40 (46.5)	40 (46.5)
Female	46 (53.5)	46 (53.5)
Socioeconomic class		
Upper	20 (23.2)	64 (74.4)
Middle	30 (34.9)	15 (17.5)
Lower	36 (41.9)	7 (8.1)
Age at sickle cell diagnosis		
<1 year	11 (12.8)	---
1–5 year	69 (80.2)	---
>5 year	6 (7.0)	---
Routine medications		
Folic acid + Proguanil	74 (86.0)	---
Folic acid + Proguanil + Hydroxyurea	12 (14.0)	---
Steady-state PCV (%)		
<18	4 (4.7)	---
18–24	62 (72.1)	---
>24	20 (23.3)	---
Mean ± SD	22.8 ± 2.8	---
Frequency of crisis in the preceding year		
None	3 (3.5)	---
1–2	71 (82.6)	---
≥3	12 (13.9)	---
Previous blood transfusion(s)		
Yes	62 (72.1)	7 (8.1)
No	24 (27.9)	79 (91.9)
Body mass index (BMI) classification		
Normal	61 (70.9)	63 (73.2)
Thinness	19 (22.1)	4 (4.7)
Severe thinness	5 (5.8)	0 (0.0)
Overweight	1 (1.2)	17 (19.8)
Obese	0 (0.0)	2 (2.3)
Mean BMI (Kg/m^2^)	16.3 ± 1.9	19.8 ± 1.3
Blood pressure (Mean ± SD)		
Systolic BP	97.0 ± 9.0	103.4 ± 8.1
Diastolic BP	57.8 ± 8.9	59.3 ± 7.0

BP- Blood pressure; SD- Standard deviation; PCV- packed cell volume

### Laboratory findings of the study participants

The mean serum cystatin C levels for the SCA and control groups were 0.872 ± 0.3 (95% confidence interval [CI], 0.811 to 0.933) and 0.922 ± 0.2 (95% CI, 0.88 to 0.962), respectively. The mean eGFR of the participants based on age categories is shown in Table 2, in which there was a significant difference in the means among children older than 10 years.

**Table 2:** Mean eGFR of the study participants

Age group (Years)	Frequency	eGFR (mL/min/1.73m^2^)		T test	p value
		Subject Mean ± SD	Control Mean ± SD		
1–5	30	123 ± 47	113 ± 37	0.932	0.355
6–10	38	113 ± 43	105 ± 34	0.839	0.404
10–14	18	133 ± 55	98 ± 17	2.560	0.015^*^
All ages	86	121 ± 47	106 ± 33	2.284	0.024^*^

SD- Standard deviation; *- Significance level (i.e. p < 0.05)

While 22.1% of children with SCA had eGFR levels below 90mL/min/1.73m^2^, approximately one-fifth of them had values greater than 140mL/min/1.73m^2^ as shown in Table 3.

**Table 3:** Comparison by eGFR categories of the study groups

eGFR classification	Subject (%) n = 86	Control (%) n = 86	Exact test	*p*-value
>140	17 (19.8)	6 (7.0)	8.310	0.039^*^
90–140	50 (58.1)	57 (66.3)		
60–89	14 (16.3)	21 (24.4)		
45–59	4 (4.6)	2 (2.3)		
30–45	1 (1.2)	0 (0.0)		
<30	0 (0.0)	0 (0.0)		

*- Significance level (i.e. p < 0.05)

### Relationship of some clinical findings in the study subjects with eGFR

There was a significant relationship between peripheral oedema and eGFR of the subjects as two children with peripheral oedema had eGFR values of less than 90mL/min/1.73m^2^. Additionally, a child on hydroxyurea had a low eGFR (Table 4).

**Table 4:** Relationship between some clinical findings and eGFR of the study subjects

Variable	eGFR (mL/min/1.73m^2^)		χ^2^	*df*	p-value
	≥ 90	< 90			
**Peripheral oedema**					
Present	0 (0.0)	2 (100.0)			
Absent	67 (79.8)	17 (20.2)	5.662	1	0.017^*^
**Frequency of crisis**					
None	3 (100.0)	0 (0.0)			
1–2	55 (77.5)	16 (22.5)			
≥3	9 (75.0)	3 (25)	0.255	2	0.993
**Steady-state PCV**					
≤18	4 (100.0)	0 (0.0)			
18–24	54 (79.4)	14 (20.6)			
>24	9 (64.3)	5 (35.7)	1.849	2	0.763
**Blood pressure**					
Low	35 (76.1)	11 (23.9)			
Normal	29 (82.9)	6 (17.1)			
Elevated BP	3 (60.0)	2 (40.0)			
Hypertension	0 (0.0)	0 (0.0)	6.786	3	0.327
**Routine medication**					
Folic acid + Proguanil	57 (77.0)	17 (23.0)			
Folic acid + Proguanil + Hydroxyurea	11 (91.7)	1 (8.3)	1.337	1	0.248

χ^2^ - Chi-square; *df*- the degree of freedom; *- Significance level (i.e. p < 0.05)

**Fig. 1:**
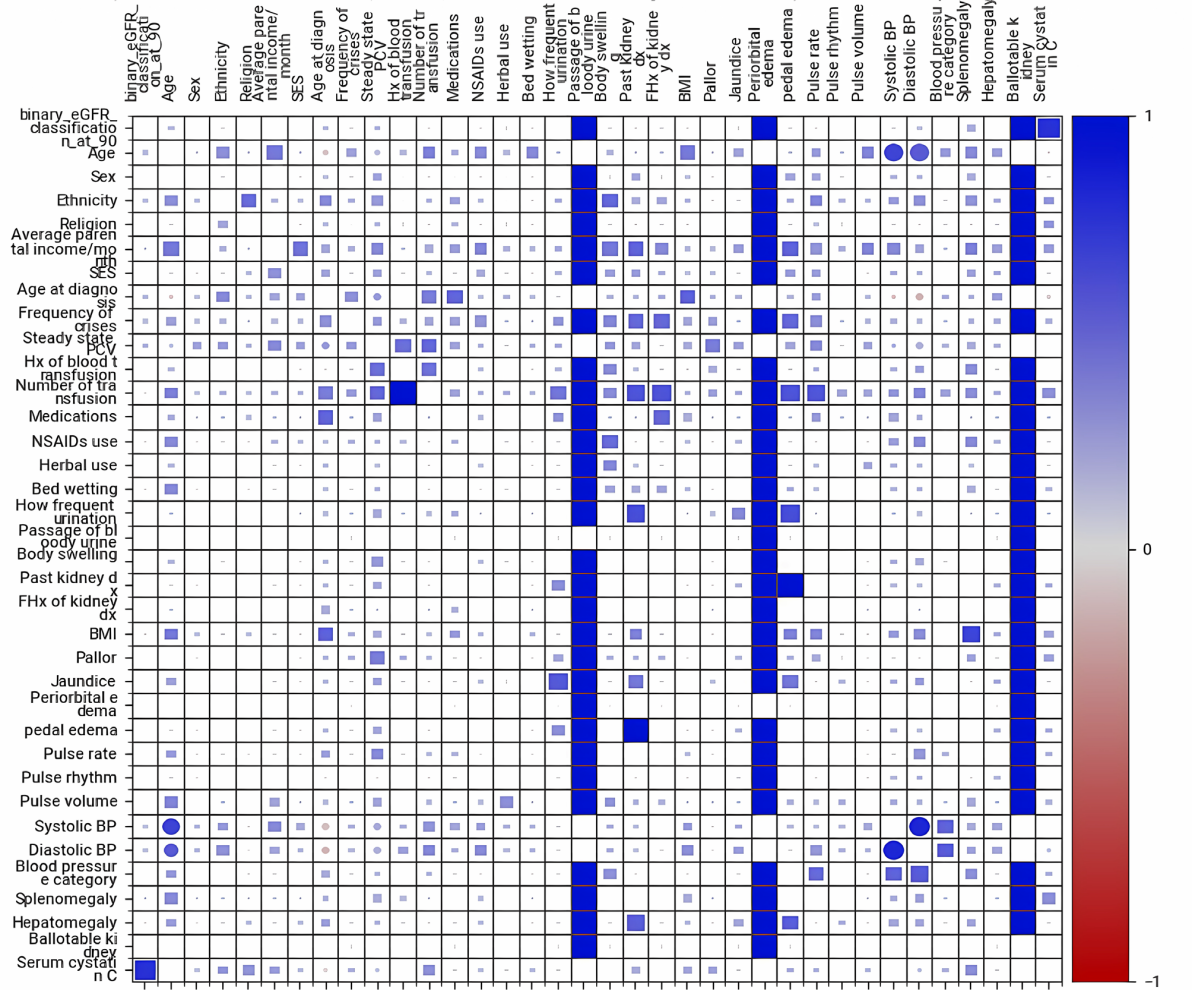
Heatmap showing correlations between test variables. Binary eGFR classification refers to eGFR classification into < 90 and ≥ 90mL/min/1.73m^2^; Hx- History; FHx- Family history; SES- Socioeconomic status; dx- Disease; BP- blood pressure

There was no significant association (uncertainty coefficient) between eGRF (categorised as < 90 and ≥ 90mL/min/1.73m^2^) and other demographic and clinical variables tested. This is shown in the first column of Fig. 1.

Whereas variables such as history of previous passage of bloody urine, NSAIDs use by participants and periorbital oedema are not associated with decreased eGFR of < 90ml/min/1.73m^2^, the steady-state PCV of the subject, age at diagnosis, and systolic and diastolic blood pressures correlate marginally with it (Table 5).

**Table 5:** Association of test variables with eGFR < 90ml/min/1.73m^2^

Variable	Uncertainty coefficient (0 to 1)	Correlation ratio (0 to 1)
Previous haematuria	1.00	-
Periorbital oedema	1.00	-
Frequency of crisis	0.03	-
Bedwetting	0.02	-
NSAIDs use	0.02	-
Age	-	0.08
Age at diagnosis	-	0.09
Systolic BP	-	0.06
Diastolic BP	-	0.06
Steady-state PCV	-	0.11

BP- blood pressure; NSAIDs- nonsteroidal anti-inflammatory drugs; PCV- packed cell volume

**Fig. 2:**
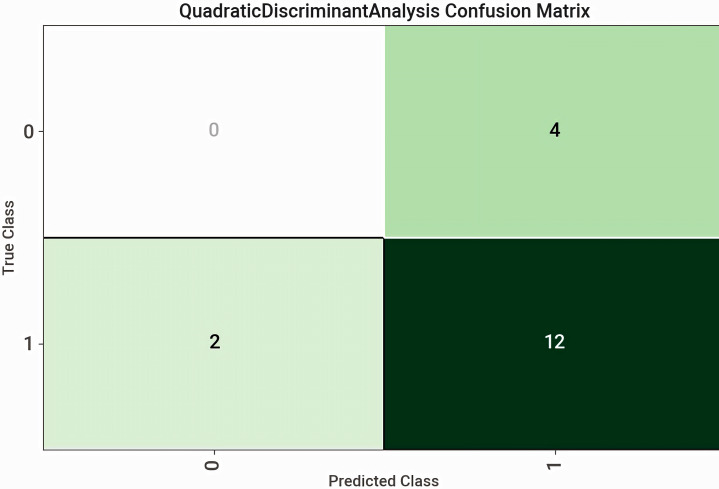
Quadratic discriminant analysis confusion matrix

Modelling was run on all test data except ethnicity due to its unbalanced nature, and serum cystatin C which was used to generate eGFR via equation. The model with the highest accuracy and precision was the Quadratic Discriminant Analysis (QDA) at 78%, area under the curve (AUC) at 50%, recall at 100% and harmonic mean (F1) at 87.5%. The QDA confusion matrix is shown in Fig. 2.

## Discussion

The glomerular filtration rate (GFR) is a dependable indicator of renal function, as it is widely regarded as the best overall index both in health and disease [16]. This study found a significant difference in the estimated GFR between children with SCA and their HbAA counterparts. Overall, the prevalence of “decreased GFR”, defined as eGFR less than 90mL/min/1.73m^2^ in children with SCA, was comparable among the study participants but was more common among children in the control group. Furthermore, factors such as the steady-state PCV, blood pressure and the occurrence of peripheral oedema in children with SCA are not associated with decreased GFR.

Our finding of a significant difference in eGFR among the study groups in this study is comparable to previous studies using different renal biomarkers [17, 18]. This could be a consequence of glomerular hyperfiltration in patients with SCA, especially in the younger age group, which predominated in this study. Hyperfiltration occurs as early as the infancy period [19, 20] and results from repeated sickling and sludging in the renal medullary vessels, producing ischemia and infarction secondarily leading to the release of vasoactive prostaglandins that mediate the increased renal cortical blood flow, thereby causing an increased GFR [20, 21]. The finding of a significant difference in the mean eGFR is, however, not in consonance with some previous studies, probably due to differences in methodology [11, 22–24]. We found a higher supernormal GFR in children with SCA relative to the controls. A markedly higher rate was reported by Aygun *et* al [25]. Generally, the GFR is known to increase in younger patients with SCA, plateaus until the late adolescent age and subsequently begins to fall during adulthood [21, 26].

The prevalence of “decreased GFR” in this study was 22.1% and 1.2% for the SCA and HbAA groups, using a cutoff of 90mL/min/1.73m^2^ and 60mL/min/1.73m^2^ respectively. Using a similar cutoff value, this value of 22.1% in SCA children is similar to that reported by Ajite et al. in Nigeria [27]. McPherson Yee et al. reported a CKD prevalence of 26.5% in their study [28]. Similarly, CKD was reported in 40.8% of HbSS participants in a study by Ephraim et al. in Ghana [29]. In contrast to this current study, the seemingly higher prevalence reported in both studies could be substantiated by the fact that, in addition to the difference in biomarkers used, their participants’ ages extend to late adolescence and early adulthood. The majority of their subjects with CKD had GFR that was greater than 90mL/min/1.73m^2^ but with evidence of kidney damage (albuminuria) given a large prevalence of stage I CKD. Although derived for use in children with CKD, the Schwartz method of GFR estimation is known to overestimate GFR [30]. The bias for overestimation is likely to be greater in children with SCA due to increased tubular secretion of creatinine as a result of hyperfiltration and decreased muscle mass. Low GFR is common during adult life as a result of glomerulosclerosis due to hyperfiltration-mediated injury. A report by Schmitt et al. indicated decreased glomerular size selectivity with enhanced macromolecule movement across filtration barriers, an increase in the ultrafiltration coefficient and loss of evidence of reversible changes as the major contributors to the decrease in GFR [31].

Clinical manifestations of CKD include hypertension, worsening anaemia and peripheral oedema. The measured systolic and diastolic blood pressures in this current study were significantly lower in children with SCA relative to the controls. This is similar to most previous work on the subject, with only a few documenting hypertension among their participants [27, 32]. The relatively low blood pressure is attributable to the low systemic vascular resistance seen in SCA patients as a result of endogenous vasodilators such as prostaglandins and nitric oxide that are recruited to enhance tissue oxygenation [33]. Increased cardiac output and aberrant glomerular vascular responses have also been implicated [34]. It could also be the result of a renal tubular defect that is responsible for increased sodium and water excretion, which may blunt the plasma volume expansion that is necessary for sustained hypertension and thus promote lower arterial pressures in a similar version with patients with salt-losing nephritis [35]. However, the precise reasons for the relative hypotension are not well understood.

Our study found elevated blood pressure in 5.8% of the SCA group which was in discordance with the 16.7% reported by Bodas et al. [36]. Given the nature of sickle cell vasculopathy, it is particularly surprising that secondary hypertension due to renal vascular obstruction or thrombosis is not a common finding in children with SCA. Hatch et al. suggested that although renin may be elevated, there is a decreased responsiveness to the vasoconstrictor effects of angiotensin in these patients [33]. The finding of elevated blood pressure predominantly among the control group (i.e. 12.8% vs. 5.8%) in this index study further supports the relatively lower blood pressure in children with SCA.

Anaemia is a well-known occurrence in people with SCA. Despite the majority of our participants having mild-to-moderate anaemia, this neither predicts nor correlates with a “decreased GFR”. In the same vein, peripheral oedema does not predict a decreased GFR among our participants. The occurrence of peripheral oedema in SCA is a function of the degree of glomerular damage, and its emergence appears to be similar to the development of hypertension indicating that proteinuria causing oedema is a feature of progressive renal damage [37].

### Strengths and limitations

Our study recruited SCA children as young as 1 year of age as only a few studies have involved this category of patients. This allowed us to investigate their eGFR characteristics relative to those of grown-up children. We also recruited a one-to-one age-appropriately paired control group to counter possible selection bias that could have untoward effects on the conclusion of the study.

In addition to the cost and scarce availability of cystatin C test kits in the country, our participants would have been better followed up at 3 months after enrollment for adequate and appropriate diagnosis of CKD. This study did not consider urine examination in addition to the serum cystatin C assay and could constitute another limitation. This would, however, serve as a template for further studies.

## Conclusions

Hyperfiltration manifesting as supernormal GFR is common among children with SCA. Likewise, the prevalence of decreased GFR is high. Clinical features such as age at diagnosis, frequency of crises, haematuria, blood pressure, and peripheral oedema were marginally associated with decrease GFR in children with SCA. It is, however, pertinent that the presence of any of these features should raise suspicion for further renal evaluation for early diagnosis of renal insufficiency.

## Data Availability

The datasets used and analysed during the current study are available from the corresponding author upon reasonable request.

## Abbreviations

CI: Confidence interval
CKD: Chronic kidney disease
ELISA: Enzyme-linked immunosorbent assay
FMC: Federal Medical Centre
GFR: Glomerular filtration rate
HbAA: Normal haemoglobin
HbSS: Homozygous sickle haemoglobin
OR: Odds ratio
SCA: Sickle cell anaemia
SCD: Sickle cell disease
SCN: Sickle cell nephropathy
